# Real world ocean rogue waves explained without the modulational instability

**DOI:** 10.1038/srep27715

**Published:** 2016-06-21

**Authors:** Francesco Fedele, Joseph Brennan, Sonia Ponce de León, John Dudley, Frédéric Dias

**Affiliations:** 1 School of Civil & Environmental Engineering, Georgia Institute of Technology, Atlanta, Georgia 30332, USA; 2 School of Electrical & Computer Engineering, Georgia Institute of Technology, Atlanta, Georgia 30332, USA; 3 University College Dublin, School of Mathematics and Statistics, Belfield, Dublin 4, Ireland; 4 Institut FEMTO-ST CNRS-Université de Franche-Comté UMR 6174 France

## Abstract

Since the 1990s, the modulational instability has commonly been used to explain the occurrence of rogue waves that appear from nowhere in the open ocean. However, the importance of this instability in the context of ocean waves is not well established. This mechanism has been successfully studied in laboratory experiments and in mathematical studies, but there is no consensus on what actually takes place in the ocean. In this work, we question the oceanic relevance of this paradigm. In particular, we analyze several sets of field data in various European locations with various tools, and find that the main generation mechanism for rogue waves is the constructive interference of elementary waves enhanced by second-order bound nonlinearities and not the modulational instability. This implies that rogue waves are likely to be rare occurrences of weakly nonlinear random seas.

According to the most commonly used definition, rogue waves are unusually large-amplitude waves that appear from nowhere in the open ocean. Evidence that such extremes can occur in nature is provided, among others, by the Draupner and Andrea events, which have been extensively studied over the last decade^1^,^2^,^3^,^4^,^5^,^6^. Several physical mechanisms have been proposed to explain the occurrence of such waves^7^, including the two competing hypotheses of nonlinear focusing due to third-order quasi-resonant wave-wave interactions^8^, and purely dispersive focusing of second-order non-resonant or bound harmonic waves, which do not satisfy the linear dispersion relation^9^,^10^.

In particular, recent studies propose third-order quasi-resonant interactions and associated modulational instabilities^11^,^12^ inherent to the Nonlinear Schrödinger (NLS) equation as mechanisms for rogue wave formation^3^,^8^,^13^,^14^,^15^. Such nonlinear effects cause the statistics of weakly nonlinear gravity waves to significantly differ from the Gaussian structure of linear seas, especially in long-crested or unidirectional (1D) seas^8^,^10^,^16^,^17^,^18^,^19^. The late-stage evolution of modulation instability leads to breathers that can cause large waves^13^,^14^,^15^, especially in 1D waves. Indeed, in this case energy is ‘trapped’ as in a long wave-guide. For small wave steepness and negligible dissipation, quasi-resonant interactions are effective in reshaping the wave spectrum, inducing large breathers via nonlinear focusing before wave breaking occurs^16^,^17^,^20^,^21^. Consequently, breathers can be observed experimentally in 1D wave fields only at sufficiently small values of wave steepness^20^,^21^,^22^. However, wave breaking is inevitable when the steepness becomes larger: ‘breathers do not breathe’^23^ and their amplification is smaller than that predicted by the NLS equation, in accord with theoretical studies^24^ of the compact Zakharov equation^25^,^26^ and numerical studies of the Euler equations^27^,^28^.

Typical oceanic wind seas are short-crested, or multidirectional wave fields. Hence, we expect that nonlinear focusing due to modulational effects is diminished since energy can spread directionally^16^,^18^,^29^. Thus, modulation instabilities may play an insignificant role in the wave growth especially in finite water depth where they are further attenuated^30^.

Tayfun^31^ presented an analysis of oceanic measurements from the North Sea. His results indicate that large waves (measured as a function of time at a given point) result from the constructive interference (focusing) of elementary waves with random amplitudes and phases enhanced by second-order non-resonant or bound nonlinearities. Further, the surface statistics follow the Tayfun^32^ distribution^32^ in agreement with observations^9^,^10^,^31^,^33^. This is confirmed by a recent data quality control and statistical analysis of single-point measurements from fixed sensors mounted on offshore platforms, the majority of which were recorded in the North Sea^34^. The analysis of an ensemble of 122 million individual waves revealed 3649 rogue events, concluding that rogue waves observed at a point in time are merely rare events induced by dispersive focusing. Thus, a wave whose crest height exceeds the rogue threshold^2^ 1.25*H*_*s*_ occurs on average once every *N*_*r*_ ~ 10^4^ waves with *N*_*r*_ referred to as the return period of a rogue wave and *H*_*s*_ is the significant wave height. Some even more recent measurements off the west coast of Ireland^35^ revealed similar statistics with 13 rogue events out of an ensemble of 750873 individual waves and *N*_*r*_ ~ 6 · 10^4^.

To date, it is still under debate if in typical oceanic seas second-order nonlinearities dominate the dynamics of extreme waves as indicated by ocean measurements^31^,^33^, or if third-order nonlinear effects play also a significant, if not dominant, role in rogue-wave formation. The preceding provides our principal motivation for studying the statistical and physical properties of rogue sea states and to investigate the relative importance of second and third-order nonlinearities. We rely on WAVEWATCH III hindcasts and High Order Spectral (HOS) simulations of the Euler equations for water waves^36^. In our study, we consider the famous Draupner and Andrea rogue waves and the less well known Killard rogue wave^35^. The Andrea rogue wave was measured by Conoco on 9 November 2007 with a system of four Teledyne Optech lasers mounted in a square array on the Ekofisk platform in the North Sea in a water depth *d* = 74 m^4^,^5^. The metocean conditions of the Andrea wave are similar to those of the Draupner wave measured by Statoil at a nearby platform (*d* = 70 m) on 1 January 1995 with a down looking laser-based wave sensor^37^. The Killard wave was measured by ESB International on 28 January 2014 by a Waverider buoy off the west coast of Ireland in a water depth *d* = 39 m. In Table 1 we summarize the wave parameters of the three sea states in which the rogue wave occurred and we refer to the Methods section for definitions and details. As one can see, the actual crest-to-trough (wave) heights *H* and crest heights *h* meet the classical criteria^2^ *H*/*H*_*s*_ > 2 and *h*/*H*_*s*_ > 1.25 to qualify the Andrea, Draupner and Killard extreme events as rogue waves. The remainder of the paper is organized as follows. First, the probability structure of oceanic seas is presented^33^ together with the essential elements of Tayfun’s^32^ second-order theory for the wave skewness and Janssen’s^8^ formulation for the excess kurtosis of multidirectional seas^29^. Then, we present and compare second-order and third-order statistical properties of the three rogue sea states followed by an analysis of the shape of the largest waves and associated mean sea levels. In concluding, we discuss the implications of these results on rogue-wave predictions.

## Probability structure of oceanic seas

Non-resonant and resonant wave-wave interactions cause the statistics of weakly nonlinear gravity waves to significantly differ from the Gaussian structure of linear seas^8^,^10^,^16^,^17^,^18^,^38^. The relative importance of ocean nonlinearities and the increased occurrence of large waves can be measured by integral statistics such as the wave skewness *λ*_3_ and the excess kurtosis *λ*_40_ of the zero-mean surface elevation *η*(*t*):
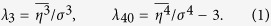


Here, overbars imply statistical averages and *σ* is the standard deviation of surface wave elevations. Here and in the following we refer to the Methods section for the definitions of the wave parameters and details.

The skewness coefficient represents the principal parameter with which we describe the effects of second-order bound nonlinearities on the geometry and statistics of the sea surface with higher sharper crests and shallower more rounded troughs^9^,^32^,^33^. The excess kurtosis comprises a dynamic component due to third-order quasi-resonant wave-wave interactions and a bound contribution induced by both second- and third-order bound nonlinearities,^9^,^10^,^32^,^33^,^39^,^40^. In order to compare the relative orders of nonlinearities, we consider the characteristic wave steepness *μ*_*m*_ = *k*_*m*_*σ*, where *k*_*m*_ is the wavenumber corresponding to the mean spectral frequency *ω*_*m*_^32^.

## Return period of a wave whose crest height exceeds a given threshold

To describe the statistics of rogue waves, we consider the conditional return period *N*_*h*_(ξ) of a wave whose crest height exceeds the threshold *h* = ξ*H*_*s*_, namely
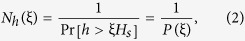
where *P*(ξ) is the probability of a wave crest height exceeding ξ*H*_*s*_. Equation (2) implies that the threshold ξ*H*_*s*_, with *H*_*s*_ = 4*σ*, is exceeded on average once every *N*_*h*_(ξ) waves.

For weakly nonlinear random seas, the probability *P* can be described by the (third-order) TF, (second-order Tayfun) T or (linear Rayleigh) R distributions. In particular^33^,
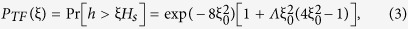
where ξ_0_ follows from the quadratic equation 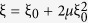
32. Here, the wave steepness *μ* = *λ*_3_/3 is of *O*(*μ*_*m*_) and it is a measure of second-order bound nonlinearities as it relates to the skewness of surface elevations^9^. The relationship *λ*_3_ = 3*μ* is originally due to Tayfun^31^, who derived it for narrowband nonlinear waves that display a vertically asymmetric profile with sharper and higher crests and shallower and more rounded troughs. As such this sort of asymmetry is also reflected in a quantitative sense in the skewness coefficient *λ*_3_ of surface elevations from the mean sea level. Although the relationship was thought to be appropriate to only narrowband waves, Fedele & Tayfun^9^ have more recently verified that it is also valid for broadband waves. In simple terms, *μ* = *λ*_3_/3 serves as a convenient relative measure of the characteristic crest-trough asymmetry of ocean waves. For narrowband (NB) waves in intermediate water depth, Tayfun^41^ derived a compact expression that reduces to the simple form *λ*_3,*NB*_ = 3*μ*_*m*_ in deep water^32^ (see Methods section for details). The parameter *Λ* in Eq. (3) is a measure of third-order nonlinearities as a function of the fourth order cumulants of the wave surface^33^. Our studies show that it is approximated by *Λ*_appr_ = 8*λ*_40_/3 (see Methods section). For second-order seas, hereafter referred to as Tayfun sea states^42^, *Λ* = 0 only and *P*_*TF*_ in Eq. (3) yields the Tayfun (T) distribution^32^
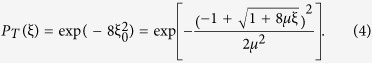


For Gaussian seas, *μ* = 0 and *Λ* = 0 and *P*_*TF*_ reduces to the Rayleigh (R) distribution
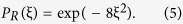


We point out that the Tayfun distribution represents an exact result for large second order wave crest heights and it depends solely on the steepness parameter defined as *μ* = *λ*_3_/3^9^. In the following, we will not dwell on wave heights^43^,^44^ as our main focus will be the statistics of crest heights in oceanic rogue sea states.

## Excess kurtosis

For third-order nonlinear random seas the excess kurtosis
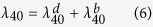
comprises a dynamic component 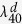 due to nonlinear quasi-resonant wave-wave interactions^8^,^40^ and a Stokes bound harmonic contribution 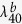
45. Janssen^45^ derived a complex general formula for the bound excess kurtosis. For narrowband (NB) waves in intermediate water depth, the formula is more compact (see Eq. (A23) in^45^ and Methods section). In deep water it reduces to the simple form 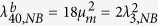
40,45,46 where *λ*_3,*NB*_ = 3*μ*_*m*_^9^,^32^,^33^. As for the dynamic component, Fedele^29^ recently revisited Janssen’s^8^ weakly nonlinear formulation for 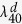. In deep water, this is given in terms of a six-fold integral that depends on the Benjamin-Feir index *BFI* and the parameter 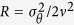, which is a dimensionless measure of the multidirectionality of dominant waves, with *ν* the spectral bandwidth and *σ*_*θ*_ the angular spreading^40^,^47^. As waves become 1D waves *R* tends to zero. Note that the *R* − values for the three rogue sea states in Table 1 range from 0.4 to 0.6.

For deep-water narrowband waves characterized by a Gaussian type directional spectrum, the six-fold integral can be reduced to a one-fold integral, so that the dynamic excess kurtosis is computed as^29^
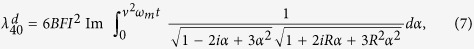
where *ω*_*m*_ is the mean spectral frequency, *ν* the spectral bandwidth, 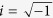 and Im(*x*) denotes the imaginary part of *x*. In the focusing regime (0 < *R* < 1) the dynamic excess kurtosis of an initially homogeneous Gaussian wave field grows, attaining a maximum at the intrinsic time scale 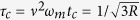. Thus, the sea state initially deviates from being Gaussian, but eventually the excess dynamic kurtosis tends monotonically to zero as energy spreads directionally, in agreement with numerical simulations^48^. The dynamic excess kurtosis maximum is well approximated by^29^
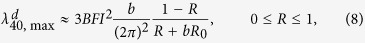
where 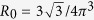 (which corrects a misprint in^29^) and *b* = 2.48. In contrast, in the defocusing regime (*R* > 1) the dynamic excess kurtosis is always negative. It reaches a minimum at *t*_*c*_ and then tends to zero over larger periods of time. In summary, the theoretical predictions indicate a decaying trend for the dynamic excess kurtosis over large times in multidirectional wave fields (*R* > 0).

In unidirectional (*R* = 0) seas, energy is ‘trapped’ as in a long wave-guide. An initially homogeneous Gaussian wave field evolves as the dynamic excess kurtosis monotonically increases toward an asymptotic non-zero value given by 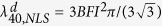 from Eq. (8)^49^. Clearly, wave energy cannot spread directionally, and quasi-resonant interactions induce nonlinear focusing and large breather-type waves initiated by modulation instability^16^,^17^,^20^,^21^,^22^,^23^,^50^. However, realistic oceanic wind seas are typically multidirectional (short-crested) and energy can spread directionally. As a result, nonlinear focusing due to modulational instability effects diminishes^16^,^18^,^29^,^51^ or becomes essentially insignificant under realistic oceanic conditions^29^. Indeed, the large excess kurtosis transient observed during the initial stage of evolution is a result of the unrealistic assumption that the initial wave field is homogeneous Gaussian whereas oceanic wave fields are usually statistically inhomogeneous both in space and time. Further, for time scales 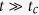, starting with initial homogeneous and Gaussian conditions becomes irrelevant as the wave field tends to a non-Gaussian state dominated by bound nonlinearities as the total kurtosis of surface elevations asymptotically approaches the value represented by the bound component^52^,^53^.

These results and conclusions hold for deep-water gravity waves. The extension to intermediate water depth *d* readily follows by redefining the Benjamin-Feir Index as 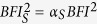 40,54, where the depth factor *α*_*S*_ depends on the dimensionless depth *k*_*m*_*d*, with *k*_*m*_ the wavenumber corresponding to the mean spectral frequency (see Methods section). In the deep-water limit *α*_*S*_ becomes 1. As the dimensionless depth *k*_*m*_*d* decreases, *α*_*S*_ decreases and becomes negative for *k*_*m*_*d* < 1.363 and so does the dynamic excess kurtosis. For the three rogue sea states under study, depth factors are less than 1 and given in Table 1 together with the associated *BFI* and *R* coefficients. From Eq. (8), the maximum dynamic excess kurtosis is of *O*(10^−3^) for all three sea states and thus negligible in comparison to the associated narrowband (NB) bound component 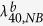 of *O*(10^−2^) (see Methods section). Hereafter, this will be confirmed further by a quantitative analysis of High Order Spectral (HOS) simulations of the Euler equations^36^.

## Results

At present, whether second-order or third-order nonlinearities play a dominant role in rogue-wave formation is a subject of considerable debate. Recent theoretical results clearly show that third-order quasi-resonant interactions play an insignificant role in the formation of large waves in realistic oceanic seas^29^. Further, oceanic evidence available so far^31^,^33^,^34^ seems to suggest that the statistics of large oceanic wind waves are not affected in any discernible way by third-order nonlinearities, including NLS-type modulational instabilities that attenuate as the wave spectrum broadens^24^. Indeed, extensive analyses of storm-generated extreme waves do not display any data trend even remotely similar to the systematic breather-type patterns observed in 1D wave flumes^10^,^31^,^33^,^34^. However, third-order bound nonlinearities may affect both skewness and kurtosis as they shape the wave surface with sharper crests and shallower troughs.

**Figure 1:**
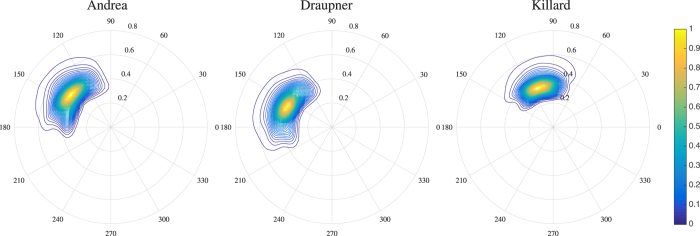
WAVEWATCH III hindcast directional wave spectra S(*ω*, *θ*) used as input for the HOS simulations. Here, *ω* is the angular frequency and *θ* the direction in degrees. (Left) Andrea, (center) Draupner, (right) Killard. The spectra have been normalized with respect to spectral peak values.

In the following we will compare second and third-order nonlinear properties of the sea states where the Draupner, Andrea and Killard rogue waves occurred, using HOS simulations of the Euler equations^36^. To do so, we first use WAVEWATCH III to hindcast the three rogue sea states. The respective directional spectra **S**(*ω*, *θ*) are shown in Fig. 1. These are used to define the initial wave field conditions for the HOS simulations–see the Methods section.

### Second-order vs third-order nonlinearities

**Figure 2:**
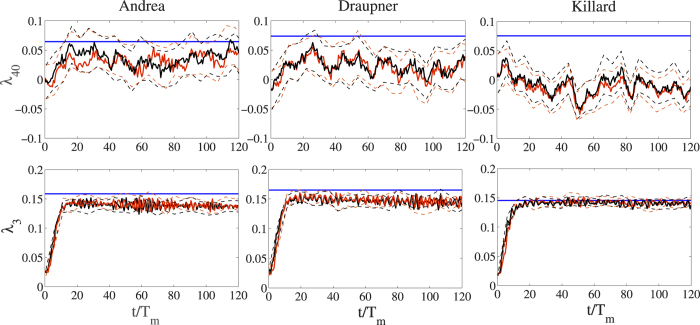
Time evolution of skewness *λ*_3_ and excess kurtosis *λ*_40_ for (left) Andrea, (center) Draupner and (right) Killard sea states; HOS second-order (black solid), HOS third-order (red solid) averages and theoretical predictions of the narrowband Tayfun skewness and Janssen excess bound kurtosis (blue solid, see Eq. (9) n Methods Section). 95% confidence bands (dashed) are also shown. Time is normalized by the mean wave period *T*_*m*_. The statistical parameters are estimated from an ensemble of 50 HOS simulations. The initial artificial transients are excluded from the ensemble averages as they are the result of a ramping function^55^ applied to the HOS equations to smoothly activate nonlinearities. See Methods section for details and definitions of wave parameters.

The time evolutions of skewness and excess kurtosis of the three simulated rogue sea states are shown in Fig. 2. Initially, the two statistics undergo a brief artificial transient of *O*(10) mean wave periods during which nonlinearities are smoothly activated by way of a ramping function^55^ applied to the HOS equations. Following this stage, we do not observe the typical overshoot beyond the Gaussian value as seen in wave tank measurements and simulations^8^,^16^,^17^,^50^. In contrast, both statistics rapidly reach a steady state as an indication that quasi-resonant wave-wave interactions due to modulation instabilities are negligible in agreement with theoretical predictions^29^. Indeed, the large-time kurtosis is mostly Gaussian for all the three sea states and there are insignificant differences between second-order and third-order HOS simulations. Further, Fig. 2 shows that the narrowband predictions slightly overestimate the observed simulated values for skewness and excess kurtosis. This is simply because narrowband approximations do not take into account the directionality and the finite bandwidth of the spectrum.

**Figure 3:**
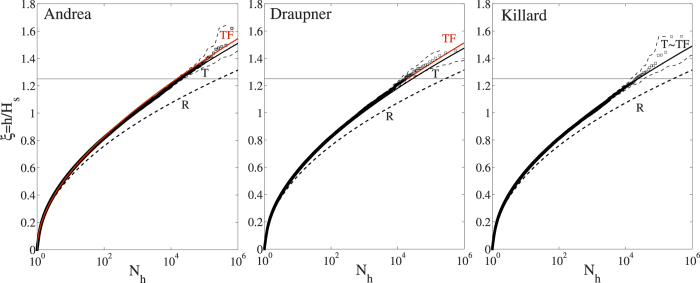
Crest height scaled by the significant wave height (ξ) versus conditional return period (*N*_*h*_) for the (left) Andrea, (center) Draupner and (right) Killard rogue sea states: HOS numerical predictions (◻) in comparison with theoretical models (T = second-order Tayfun (light solid lines), TF = third-order (red solid lines) and R = Rayleigh distributions (dark dashes)). Confidence bands are also shown (light dashes). *N*_*h*_(ξ) is the inverse of the exceedance probability *P*(ξ) = *Pr*[*h* > ξ*H*_*s*_]. Horizontal lines denote the rogue threshold 1.25*H*_*s*_^2^.

Our main conclusion is that second-order bound nonlinearities mainly affect the large-time skewness *λ*_3_ whereas excess kurtosis is smaller since it is of 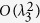
39,40 (see also Methods section). Clearly, second-order effects are the dominant factors in shaping the probability structure of the random sea state with a minor contribution of excess kurtosis effects. Such dominance is seen in Fig. 3, where the HOS numerical predictions of the conditional return period *N*_*h*_(ξ) of a crest exceeding the threshold ξ*H*_*s*_ are compared against the theoretical predictions based on the linear Rayleigh (R), second-order Tayfun (T) and third-order (TF) models from Eq. (3). In particular, *N*_*h*_(ξ) follows from Eq. (2) as the inverse 1/*P*(ξ) of the empirical probabilities of a crest height exceeding the threshold ξ*H*_*s*_. An excellent agreement is observed between simulations and the third-order TF model, which is nearly the same as the second-order T model. This indicates that second-order effects are dominant, whereas the linear Rayleigh model underestimates the empirical return periods.

For both second- and third-order nonlinearities, the return period *N*_*r*_ of a wave whose crest height exceeds the rogue threshold 1.25*H*_*s*_ is nearly 2 · 10^4^ for the Andrea, Draupner and Killard sea states. Oceanic rogue wave measurements^34^ indicate that the rogue threshold for crest heights is exceeded on average once every *N*_*r*_ ~ 10^4^ waves. Similarly, recent measurements off the west coast of Ireland^35^ yield *N*_*r*_ ~ 6 · 10^4^. In contrast, in a Gaussian sea the same threshold is exceeded more rarely and on average once every 3 · 10^5^ waves.

Note that all three rogue waves have crest heights that exceed the threshold 1.5*H*_*s*_. This is exceeded on average once every 5 · 10^5^ waves in second- and third-order seas and extremely rarely in Gaussian seas, i.e. on average once every 6 · 10^7^ waves. This implies that the three rogue wave crest events are likely to be rare occurrences of weakly second-order random seas, or Tayfun sea states^42^. Our results clearly confirm that rogue wave generation is the result of the constructive interference (focusing) of elementary waves enhanced by second-order nonlinearities in agreement with the theory of stochastic wave groups proposed by Fedele and Tayfun^9^, which relies on Boccotti’s^43^ theory of quasi-determinism^43^. Our conclusions are also in agreement with observations^9^,^10^,^31^,^33^.

### Comparison of the profiles of three rogue waves

**Figure 4:**
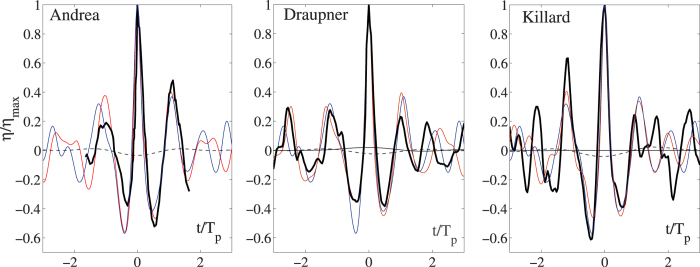
Third-order HOS simulated extreme wave profiles (red thin solid), second-order HOS profiles (blue thin solid) and mean sea levels (MSL) (thin dashed) versus the dimensionless time *t*/*T*_*p*_ for (left) Andrea, (center) Draupner and (right) Killard waves. For comparisons, measurements (thick solid) and actual MSLs (thin solid) are also shown. Note that the Killard MSL is insignificant and the Andrea MSL is not available. *T*_*p*_ is the dominant wave period (see Methods section for definitions).

For all three rogue sea states under study, the largest wave observed in the HOS simulations is now compared against the actual rogue wave measurements. Figure 4 compares the actual wave profiles (thin solid line) with the largest second-order (thin dotted-dashed line) and third-order (thick solid line) simulated waves. While there are small differences between the two orders, second-order nonlinearities are sufficient in predicting the observed profiles with sufficient accuracy.

In the same figure, the simulated mean sea level (MSL) below the crests is also shown. The estimation of the MSL follows by low-pass filtering the measured time series of the wave surface with frequency cutoff *f*_*c*_ ~ *f*_*p*_/2, where *f*_*p*_ is the frequency of the spectral peak^56^. Note that the time series must be long enough and contain at least ~200 waves for a statistically robust estimation of wave-wave interactions. In shorter time series, a set-up is observed as a manifestation of the large crest segment that extends above the adjacent lower crests. The HOS simulations give approximately the same MSL for both second- and third-order nonlinearities predicting a setdown below the large crests as expected from theory^57^. However, the observed Draupner set-up (thin line) is not reproduced by our HOS numerical simulations (see Fig. 4). We also note that the HOS MSL is close to the narrowband prediction *ST*_*NB*_ (see Table 1 and Methods section for definitions). The actual MSL for Andrea is not available, and buoy observations give neither a set-up nor a set-down for Killard.

Taylor *et al*.^58^ reported that for the Draupner wave the hindcast from the European Centre for Medium-Range Weather Forecasts shows swell waves propagating at approximately 80 degrees to the wind sea. They argued that the Draupner wave may be due to the crossing of two almost orthogonal wave groups in accord with second-order theory. This would explain the set-up observed under the large wave^56^ instead of the second-order set-down normally expected^57^. Note that the angle between the two dominant sea directions lies outside the range ~20–60 degrees where modulation instability is enhanced^59^.

**Figure 5:**
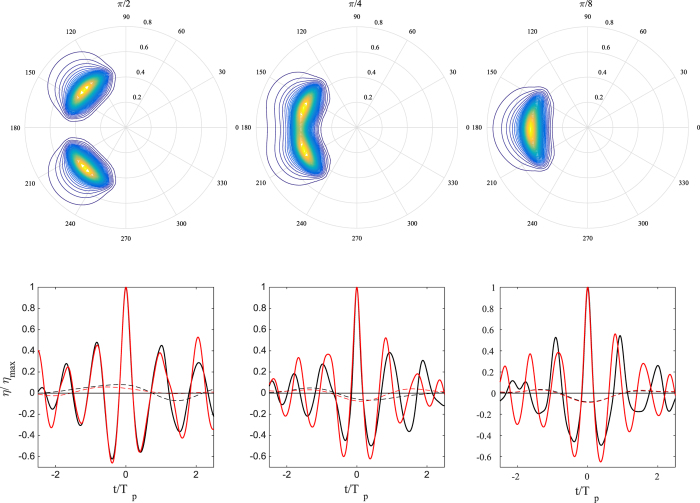
Upper row: crossing directional wave spectra S(*ω*, *θ*) computed using two identical JONSWAP spectra with Draupner spectral properties. Lower row: extreme wave profiles simulated with third order HOS (red lines) and second order HOS (black lines). In addition, the corresponding mean sea levels are shown (dashed lines). The mean sea levels are scaled by three for emphasis. Crossing angles from left to right: *π*/2, *π*/4, and *π*/8. Note that for the final case, the relatively small crossing angle results in the spectrum appearing to contain only one dominant peak.

Further studies and a high resolution hindcast of the Draupner sea state are needed to clarify if it was a crossing-seas situation as our WAVEWATCH III hindcast spectrum does not indicate so. Concerning the disagreement for the Draupner wave on the set-up, we have conducted numerical HOS experiments where the input spectrum consists of two identical JONSWAP type crossing sea states at 90 degrees. And we indeed found a set-up. As a matter of fact, whether one obtains a set-up or a set-down depends on the angle between the crossing seas. As the angle increases, the set-down turns into a set-up – see Fig. 5. However, we still find that second-order effects are dominant and third-order contributions on skewness and kurtosis, mainly due to bound nonlinearities, are negligible.

Our results are in agreement with the recent numerical simulations by Trulsen *et al*.^42^ of the crossing sea state encountered during the accident of the tanker Prestige on 13 November 2002. Puzzled by the literature on crossing seas states, they checked whether the fact that the accident occurred during a bimodal sea state with two wave systems crossing nearly at a right angle increased or not the chance of encountering a rogue wave. They concluded that the wave conditions at the time of the accident were only slightly more extreme than those of a Gaussian sea state, and slightly less extreme than those of a second-order Tayfun sea state^32^.

## Discussion

Since the 1990s, modulational instability^11^,^12^ of a class of solutions to the NLS equation has been proposed as a mechanism for rogue wave formation^3^,^8^,^13^,^14^,^15^. The availability of exact analytical solutions of 1D NLS breathers^13^ via the Inverse Scattering Transform^60^ enormously stimulated new research on rogue waves. In particular, it has been found that in 1D wave fields, the late-stage evolution of modulation instability leads to large waves in the form of breathers^13^,^14^,^15^. Indeed, in such situations energy is ‘trapped’ as in a long wave-guide, and quasi-resonant interactions are effective in inducing large breathers via nonlinear modulation before wave breaking occurs^16^,^17^,^20^,^21^. However, rogue waves in the form of breathers can be observed experimentally in 1D waves only at sufficiently small values of wave steepness (~0.01–0.09)^20^,^21^,^22^. Indeed, wave breaking is inevitable for wave steepness larger than 0.1: ‘breathers do not breathe’^23^, and their amplification is smaller than that predicted by the NLS equation, as confirmed by numerical simulations^27^,^28^.

Clearly, typical oceanic wind seas are short-crested, or multidirectional wave fields and their dynamics is more ‘free’ than the 1D ‘long-wave-guide’ counterpart. Indeed, energy can spread directionally and as a result nonlinear focusing due to modulational instability is diminished^16^,^18^,^29^. Our results suggest that in typical oceanic fields third-order nonlinearities do not play a significant role in the wave growth.

Furthermore, we found that skewness effects on crest heights are dominant in comparison to bound kurtosis contributions and statistical predictions can be based on second-order models^32^,^33^,^61^. Thus, rogue waves are likely to be rare occurrences resulting from the constructive interference (dispersive and directional focusing) of elementary waves enhanced by second order nonlinear effects in agreement with observations^9^,^10^,^31^,^33^ and with the theory of stochastic wave groups^9^. This theory about the mechanics of wave groups shows that they can be thought of as genes of a non-Gaussian sea dominated by second-order nonlinearities, when interested in the dynamics of large surface displacements. The space-time evolution of wave crests during an extreme event can be seen in the Supplementary Video S1 of the simulated Killard rogue wave sea state analyzed in this paper. We anticipate that our results may motivate similar analysis of waves over a wider distribution of heights using extensive data sets^34^.

## Methods

### Wave parameters

The significant wave height *H*_*s*_ is defined as the mean value *H*_1/3_ of the highest one-third of wave heights. It can be estimated either from a zero-crossing analysis or more easily from the wave omnidirectional spectrum 
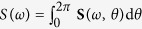
 as *H*_*s*_ ≈ 4*σ*, where 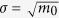 is the standard deviation of surface elevations, *m*_*j*_ = ∫*S*(*ω*)*ω*^*j*^d*ω* are spectral moments and **S**(*ω*, *θ*) is the directional wave spectrum.

The dominant wave period *T*_*p*_ = 2*π*/*ω*_*p*_ refers to the frequency *ω*_*p*_ of the spectral peak. The mean zero-crossing wave period *T*_0_ is equal to 2*π*/*ω*_0_, with 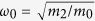. The associated wavelength *L*_0_ = 2*π*/*k*_0_ follows from the linear dispersion relation 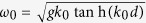, with *d* the water depth. The mean spectral frequency is defined as *ω*_*m*_ = *m*_1_/*m*_0_^32^ and the associated mean period *T*_*m*_ is equal to 2*π*/*ω*_*m*_. A characteristic wave steepness is defined as *μ*_*m*_ = *k*_*m*_*σ*, where *k*_*m*_ is the wavenumber corresponding to the mean spectral frequency *ω*_*m*_^32^. The following quantitites are also introduced: *q*_*m*_ = *k*_*m*_*d*, *Q*_*m*_ = tanh*q*_*m*_, the phase velocity *c*_*m*_ = *ω*_*m*_/*k*_*m*_, the group velocity *c*_*g*_ = *c*_*m*_[1 + 2*q*_*m*_/sinh(2*q*_*m*_)]/2. The spectral bandwidth 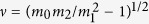 gives a measure of the frequency broadening. The angular spreading is estimated as 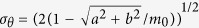, where 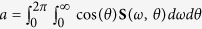 and 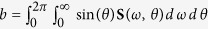
62. Note that 
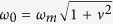
.

The parameter *Λ* = *λ*_40_ + 2*λ*_22_ + *λ*_04_ is a measure of third-order nonlinearities and is a function of the fourth order cumulants *λ*_*nm*_ of the wave surface *η* and its Hilbert transform 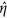
33. In particular, 
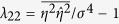
 and 
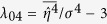
. In practice, *Λ* is usually approximated solely in terms of the excess kurtosis as *Λ*_appr_ = 8*λ*_40_/3 by assuming the relations between cumulants^49^ *λ*_22_ = *λ*_40_/3 and *λ*_04_ = *λ*_40_. These, to date, have been proven to hold for linear and second-order narrowband waves only^39^. For third-order nonlinear seas, our numerical studies indicate that *Λ* ≈ *Λ*_appr_ within a 3% relative error in agreement with observations^19^,^63^.

The wave steepness *μ* = *λ*_3_/3 relates to the wave skewness *λ*_3_ of surface elevations. For narrowband (NB) waves in intermediate water the wave skewness^41^ and bound excess kurtosis^45^
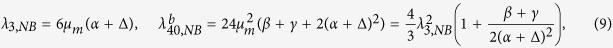
where
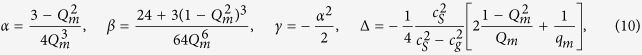
with 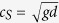 the phase velocity in shallow water. The wave-induced set-down or mean sea level variation below a crest of amplitude *h* is *ST*_*NB*_ = Δ*h*^2 ^45. In deep water,
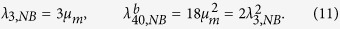


Note that Eq. (9) are not valid in small water depth as second and third-order terms of the associated Stokes expansion can be larger than the linear counterpart (see Eq. (A18) in^45^). To be valid, the constraints *αμ*_*m*_ ≤ 1 and *βμ*_*m*_/*α* ≤ 1 must hold. And indeed they are satisfied for the three rogue sea states under study. The depth factor *α*_*S*_ depends on *k*_*m*_*d* through of a lengthy expression, which is not reported here for the sake of simplicity – see Janssen and Onorato^54^.

### Brief description of WAVEWATCH III and hindcast validation

WAVEWATCH III^62^,^64^ is a third generation wave model developed at NOAA/NCEP that solves the spectral energy action balance equation with a source function representing the wind input, wave-wave interactions and the wave energy dissipation due to diverse processes. The configuration of the model was set to solve the balance equation from a minimum frequency of 0.0350 Hz up to 0.5552 Hz for 36 directional bands and 30 frequencies. A JONSWAP spectrum was set as an initial condition at every grid point. We used the wind input fields from the NOAA Climate Forecast System Reanalysis (CFSR)^64^.

### Higher Order Spectral Method

The HOS method is a numerical pseudo spectral method to solve the Euler equations governing the dynamics of incompressible fluid flow at a desired level of nonlinearity. In particular, the time evolution of the free surface of the fluid, *η*(*x*, *y*, *t*), and the associated velocity potential *ψ*(*x*, *y*, *t*) evaluated on the free surface are obtained. The method was independently developed in 1987 by Dommermuth & Yue^36^ and West *et al*.^65^. Within the present work, West *et al*.’s version is employed. Tanaka^66^ provides a thorough description of the method.

Initial conditions for the potential *ψ* and surface elevation *η* are obtained from the directional spectrum as an output of WAVEWATCH III. In the wavenumber domain, the Fourier transform 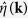 of *η* is constructed as *S*(**k**)exp(*iβ*), where *β* is normally distributed over [0, 2*π*]. Similarly, the Fourier transform 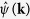 of *ψ* is obtained via linear wave theory, and finally an inverse Fourier transform is applied. The numerical simulation is performed using 1024 × 1024 Fourier modes and over a time scale 
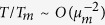
, where *μ*_*m*_ represents a characteristic wave steepness defined above. A low-pass filter is applied to avoid numerical blow-up.

Finally, we note that the use of the WAVEWATCH III model combined with HOS simulations may prove useful in assessing recently proposed techniques for rogue wave predictability based on chaotic time series analysis^67^,^68^ and third-order probabilistic models of unexpected wave extremes^69^.

## Additional Information

**How to cite this article**: Fedele, F. *et al*. Real world ocean rogue waves explained without the modulational instability. *Sci. Rep.* **6**, 27715; doi: 10.1038/srep27715 (2016).

## Supplementary Material

Supplementary Information

Supplementary Movie 1

## Figures and Tables

**Table 1:** Wave parameters and various statistics of the simulated sea states labelled Andrea, Draupner and Killard.

	**Andrea**	**Draupner**	**Killard**
Significant wave height *H*_*s*_ [m]	10.0	11.2	11.4
Dominant wave period *T*_*p*_ [s]	14.3	15.0	17.2
Mean zero-crossing wave period *T*_0_ [s]	11.6	12.1	14.0
Mean wavelength *L*_0_ [m]	209	219	268
Depth *d* [m], *k*_0_*d*	74, 2.23	70, 2.01	58, 1.36
Spectral bandwidth *ν*	0.35	0.36	0.37
Angular spreading *σ*_*θ*_	0.37	0.39	0.34
Parameter 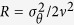 40	0.56	0.59	0.42
Benjamin Feir Index *BFI* in deep water^8^	0.24	0.23	0.18
Depth factor *α*_*S*_^40^	0.31	0.36	0.04
Tayfun NB skewness *λ*_3,*NB*_^41^	0.159	0.165	0.145
Mean skewness *λ*_3_ from HOS simulations	0.141	0.146	0.142
Maximum NB dynamic excess kurtosis 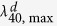 29	2.3 · 10^−3^	2.1 · 10^−3^	2.7 · 10^−4^
Janssen NB bound excess kurtosis 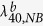 45	0.065	0.074	0.076
Mean excess kurtosis *λ*_40_ from HOS simulations	0.041	0.032	−0.011
Janssen NB setdown *ST*_*NB*_/*H*_*s*_^45^, predicted HOS setdown	0.12, 0.08	0.1, 0.11	0.1, 0.07
Maximum crest height *h*/*H*_*s*_: observed, numerical	1.63, 1.71	1.55, 1.54	1.44, 1.57
Maximum crest-to-trough (wave) height *H*/*H*_*s*_: observed, numerical	2.30, 2.51	2.10, 2.23	2.00, 2.28
Maximum trough-to-crest (wave) height *H*/*H*_*s*_: observed, numerical	2.49, 2.67	2.15, 2.09	2.32, 2.29

The Killard rogue wave occurred on a water depth of 39 m, however the hincast input spectrum could only be computed at an averaged water depth of 58 m. Statistical parameters are from an ensemble of 50 HOS simulations of sea states. We refer to the Methods section for the definitions of the wave parameters. Note that two wave heights are given for each wave: the zero-downcrossing value (crest to trough) and the zero-upcrossing value (trough to crest).
