# Supplementary material for: Real world ocean rogue waves explained without the modulational instability
Source: Sci Rep. 2016 Jun 21;6:27715. doi: 10.1038/srep27715 (PMC4914928; doi:10.1038/srep27715)
Supplement: Supplementary Information [file srep27715-s1.pdf]

**SREP-16-09187A**

**Real world ocean rogue waves explained without the modulational instability**

*Francesco Fedele, Joseph Brennan, Sonia Ponce de Leon, John Dudley, and Frederic Dias*

VIDEO S1

Simulated Killard rogue wave sea state
